# Gaze following: A socio-cognitive skill rooted in deep time

**DOI:** 10.3389/fpsyg.2022.950935

**Published:** 2022-12-02

**Authors:** Claudia Zeiträg, Thomas Rejsenhus Jensen, Mathias Osvath

**Affiliations:** Department of Philosophy and Cognitive Science, Lund University, Lund, Sweden

**Keywords:** gaze, evolution, social cognition, social information, orienting

## Abstract

Social gaze has received much attention in social cognition research in both human and non-human animals. Gaze following appears to be a central skill for acquiring social information, such as the location of food and predators, but can also draw attention to important social interactions, which in turn promotes the evolution of more complex socio-cognitive processes such as theory of mind and social learning. In the past decades, a large number of studies has been conducted in this field introducing differing methodologies. Thereby, various factors influencing the results of gaze following experiments have been identified. This review provides an overview of the advances in the study of gaze following, but also highlights some limitations within the research area. The majority of gaze following studies on animals have focused on primates and canids, which limits evolutionary interpretations to only a few and closely related evolutionary lineages. This review incorporates new insights gained from previously understudied taxa, such as fishes, reptiles, and birds, but it will also provide a brief outline of mammal studies. We propose that the foundations of gaze following emerged early in evolutionary history. Basic, reflexive co-orienting responses might have already evolved in fishes, which would explain the ubiquity of gaze following seen in the amniotes. More complex skills, such as geometrical gaze following and the ability to form social predictions based on gaze, seem to have evolved separately at least two times and appear to be correlated with growing complexity in brain anatomy such as increased numbers of brain neurons. However, more studies on different taxa in key phylogenetic positions are needed to better understand the evolutionary history of this fundamental socio-cognitive skill.

## Summary

- Studies on gaze following and object choice have traditionally lacked a phylogenetic focus.
- Comparisons between species are especially difficult due to different methodologies used over time.
- A variety of often disregarded factors can potentially impact the results of gaze following experiments.
- Hypotheses about the evolutionary roots of gaze following are possible through comparing this skill in distantly related species.
- Gaze following into the distance appears to be a conserved cognitive trait shared among at least all amniotes, possibly even all vertebrates.
- Geometrical gaze following seems to have evolved convergently in mammals and birds.
- More sophisticated gaze following skills appear to be the result of increased neuronal numbers.
- Non-avian dinosaurs likely already followed others’ gazes.

## Introduction

Sociality in non-human animals takes many forms, ranging from solitary species that meet only to mate, to species living in complex societies. Truly solitary species are rare, as a minimal degree of sociality can be found in many species, at least in the sexually reproductive ones. Species with varying degrees of sociality face different challenges. Consequently, species vary in their socio-cognitive repertoires.

Social interactions rely on the transfer and use of social information. Such information can either be conveyed intentionally through communicative signals or be produced inadvertently. For example, an individual’s presence in a certain location can inform others about food sources and risk of predation. Social information enables the receiver to optimize decisions, and therefore the ability to use such information is adaptive (Morand-Ferron et al., 2010).

Due to the value of social information, various functions have evolved to facilitate its use. One way of acquiring social information is to observe what others are looking at. In this way, one can use others’ visual attention to gather information about the surroundings that would have otherwise remained elusive. The ability to co-orient with others’ gaze directions is called gaze following (Butterworth and Jarrett, 1991).

The advantages of following others’ gazes are numerous and range from gathering information about the location of predators or food sources, to drawing attention to important social interactions (Tomasello et al., 1998). Eavesdropping on social interactions promotes knowledge of third-party relationships and can be used in tactical deception or observational learning (Emery, 2000). Arguably, observational learning would be difficult without gaze following, as the gaze draws attention to central affordances in a task.

Gaze following is an important fundamental skill to study, when uncovering some of the evolutionary roots of social cognition. The presence of gaze following in various distantly related species implies an origin in deep evolutionary time. This quick and easy way of gathering social information might have been the starting point for the evolution of more complex socio-cognitive skills such as empathy and theory of mind (Emery, 2000; Shepherd and Platt, 2008).

Despite gaze following appearing to be a widespread skill, the majority of animal studies have focused on primates and canids. This limits evolutionary interpretations. To better understand the evolution of social cognition in general, and of gaze following in particular, it is necessary to expand research efforts to more distantly related lineages. In recent years, the interest in such taxa has increased and a number of new studies, especially on reptiles and birds, provide insights into the evolution of gaze following.

This review describes general ideas and methodologies of the research field. It also discusses limitations and describes various factors potentially impacting the results of gaze following studies in non-human animals with the aim of improving future experimental designs.

With respect to the evolution of gaze following, this review highlights recent advances in the research on the Sauropsida lineage (reptiles and birds). Though still scarce, new evidence from this lineage is important for understanding deep evolution. We also provide an outline of studies on mammals, including human and non-human primates.

## The roots of gaze following research: Developmental psychology

Co-orienting with others’ gazes is a fundamental part of human social cognition. A classic example is when someone in a crowd suddenly looks up and scans the sky. People around will shortly start looking up as well, seeking for the object of interest.

Humans are skilled gaze followers from an early age. In fact, gaze following has first been studied within the discipline of developmental psychology. The earliest insights stem from a study by Scaife and Bruner (1975) that found that human infants start following head directions at 2–4 months, and that all children have developed this ability by 11 months.

It was later shown that already newborns are sensitive to others’ gazes (Batki et al., 2000; Farroni et al., 2002) and have a preference for direct gaze (Farroni et al., 2002). Gaze cueing, where observers orient towards an object in the direction of another’s gaze, also appears to be present in newborns (Farroni et al., 2004).

The ontogenetic onset of gaze following has later been pushed back to 3–6 months (e.g., Butterworth and Jarrett, 1991; Perra and Gattis, 2010). The extent to which infants can modulate their early gaze following responses remains unclear. Senju and Csibra (2008) showed that gaze following of 6-month-old infants depends on ostentive signals such as eye contact or addressing the child. Gredebäck et al. (2018), however, found that infants of the same age responded to gaze cues with and without ostentive signals. More recently, Ishikawa and Itakura (2019) demonstrated that arousal facilitates infants’ gaze following responses, which can in turn be heightened through ostentive signals. It should, however, be noted that the children in this study were between 9 and 10 months old. The ability to modulate gaze following responses based on arousal might thus develop later than spontaneous co-orientations.

These early gaze following responses are, however, not very precise. Infants can only reliably identify the target of observed attention at 12 months (Farroni et al., 2004; Moore, 2008). Moreover, children younger than 10 months follow the head direction of a demonstrator with open and closed eyes without distinction, implying a developmental shift in understanding referentiality of looking around that time (Woodward, 2003; Brooks and Meltzoff, 2005; Csibra and Volein, 2008; Senju et al., 2008). Gaze following abilities are fully developed at 18 months, when the infants follow gazes outside their immediate visual field and behind themselves (Butterworth and Jarrett, 1991; for a recent review see Del Bianco et al., 2019).

The development of gaze following in human infants plays a central role in the development of other socio-cognitive skills, such as theory of mind (Brooks and Meltzoff, 2015), joint attention (Carpenter et al., 1998), and language acquisition (Baldwin, 1991; Schafer and Plunkett, 1998; Houston-Price et al., 2006), illustrating its fundamental role in human social cognition.

## Neurocognitive mechanisms of gaze following

The neurocognitive mechanisms underlying gaze following are not fully understood. The consensus is, however, that two distinct pathways guide co-orientations with observed gaze directions. Fast, reflexive co-orientations (Deaner and Platt, 2003) are mediated by an evolutionary conserved subcortical pathway (Sewards and Sewards, 2002; Johnson, 2005) providing fast, yet crude co-orienting responses to gaze. In mammals, the subcortical pathway is proposed to run from the retina to the superior colliculus, the pulvinar, and finally to the amygdala (Morris et al., 1999; Johnson, 2005; Jiang and He, 2006). In fishes, amphibians, reptiles, and birds, the optic tectum represents the homologue of the superior colliculus, while the rest of the pathway remains the same.

This subcortical pathway is, however, likely insufficient to mediate spatially sophisticated gaze following, such as tracking others’ gazes around barriers. Thus, the subcortical pathway is most likely interconnected with more nuanced cortical networks in mammals, such as the fusiform gyrus (face perception and recognition: Johnson, 2005), the exastriate body area (visual processing of the body: Downing et al., 2001) and the superior temporal sulcus (functions explained below; Shepherd, 2010). The cortical homologues responsible for more complex gaze following skills in other vertebrates remain unknown.

The superior temporal sulcus (STS) of humans and non-human primates has been found to contain cells reacting to different facial orientations (e.g., Perrett et al., 1982) and some are specifically sensitive to the direction of eye gaze (Perrett et al., 1985; Yamane et al., 1988). Hence, this structure might be important in the detection of others’ visual attention.

The STS projects onto the parietal intraparietal cortex in macaques (Harries and Perrett, 1991). The lateral part of this structure (lateral parietal area, LIP) houses “gaze mirror neurons,” i.e., neurons that fire both, when looking at a specific location and when watching someone else gazing toward the same location (Shepherd et al., 2009). Such neurons might cause attentional shifts through matching the observed gaze direction with one’s own visual attention, similar to the functioning of mirror neurons of the motor system (Rizzolatti et al., 2009). Visuo-social areas of the fusiform gyrus and STS are moreover interconnected with an extended face processing network that might further modulate gaze following responses (Ishai et al., 2005; Vuilleumier and Pourtois, 2007).

Data on cortical pathways of gaze following stem exclusively from humans and macaques. Nevertheless, it has been proposed that social processing areas might be homologous in all primate (Tootell et al., 2003; Rosa and Tweedale, 2005) and possibly other mammalian species (Kendrick et al., 2001). How other taxa, such as birds, achieve spatially sophisticated gaze following skills remains unclear and will need to be addressed in future studies.

## Factors influencing gaze following

A variety of animal species have been tested for their capacity to follow others’ gaze direction. While in human studies, a distinction is often made between following the direction of the head and shifts of eye gaze alone, most studies on animals use a head directional cue in their gaze following experiments. For that reason, in this review, when speaking of gaze following, we refer to co-orientations with head directions. For a more detailed discussion of this topic, see “Eye morphology”.

Within the great number of animal gaze following studies, many factors and inconsistent methodologies can significantly impact subjects’ performances. Two experimental paradigms are used when studying how animals use social information conveyed through gaze: (1) visual co-orientation, and (2) object choice. In the former, the animal is presented with a demonstrator looking toward a specific location in the environment and the observer’s co-orientation with the line of gaze is recorded. In object choice, subjects are tested for their capacity to use gaze cues to identify the location of food. For an overview of species tested in different experimental paradigms, see Table 1.

**Table 1:** Overview of species tested in different gaze following paradigms and corresponding references.

Species	GFD	GGF	GFBS	OC	COC	FTC	GKT	CB	References
**BIRDS**									
**Corvids**									
Clark’s nutcracker (*Nucifraga columbia)*	–	–	–	✓	–	–	–	–	Tornick et al. (2011)
Common raven (*Corvus corax)*	✓	✓	–	✘	–	–	✓	–	Bugnyar et al. (2004, 2016), Schloegl et al. (2007, 2008a,b), Bugnyar (2011)
Jackdaw (*Corvus monedula)*	✘	✘	–	✓	–	–	–	–	Schloegl et al. (2008b), von Bayern and Emery (2009)
Rook (*Corvus frugilegus)*	✓	✓	–	✘	–	–	–	–	Schloegl et al. (2008b), Schmidt et al. (2011)
**Fowl**									
Greylag goose (*Anser anser)*	✓	–	–	–	–	–	–	–	Kehmeier et al. (2011)
Red junglefowl (*Gallus gallus*)	✓	✓	–	–	–	–	–	✓	Zeiträg et al. (2022)
**Ibises**									
Northern bald ibis (*Geronticus eremita)*	✓	✘	–	–	–	–	–	–	Loretto et al. (2009)
**Palaeognaths**									
Elegant-crested tinamou (*Eudromia elegans)*	✓	✓	–	–	–	–	–	✓	Zeiträg et al. (2022)
Emu (*Dromaius novaehollandiae)*	✓	✓	–	–	–	–	–	✓	Zeiträg et al. (2022)
Rhea (*Rhea americana)*	✓	✓	–	–	–	–	–	✓	Zeiträg et al. (2022)
**Parrots**									
African gray parrot (*Psittacus erithacus)*	–	–	–	✘	–	–	–	–	Giret et al. (2009)
**Passerines**									
European starling (*Sturnus vulgaris)*	–	✓	–	–	–	–	–	–	Butler and Fernández-Juricic (2014)
**Penguins**									
African penguin (*Spheniscus demersus)*	✓	–	–	–	–	–	–	–	Nawroth et al. (2017)
**MAMMALS**									
**Canids**									
Dingo (*Canis lupus dingo)*	–	–	–	✘	–	–	–	–	Smith and Litchfield (2010)
Domestic dog (*Canis familiaris)*	✓	✓	–	✓	–	✓	–	–	Hare et al. (1998), Miklósi et al. (1998), Hare and Tomasello (1999), Agnetta et al. (2000), McKinley and Sambrook (2000), Soproni et al. (2001), Bräuer et al. (2006), Téglás et al. (2012), Maginnity and Grace (2014), Met et al. (2014), Wallis et al. (2015), Werhahn et al. (2016), Catala et al. (2017), Duranton et al. (2017), Clark and Leavens (2021)
Wolve (*Canis lupus)*	✓	✓	–	–	–	–	–	–	Range and Virányi (2011)
**Cetaceans**									
Bottlenose dolphin (*Tursiops truncatus)*	–	–	–	✓	–	–	–	–	Tschudin et al. (2001), Pack and Herman (2004)
**Felids**									
Cat (*Felis silvestris catus)*	–	–	–	✓	–	–	–	–	Pongrácz et al. (2019)
**Pinnipeds**									
South African fur seal (*Arctocephalus pusillus)*	–	–	–	✓	–	–	–	–	Scheumann and Call (2004)
Gray seal (*Halichoerus grypus)*	–	–	–	✘	–	–	–	–	Shapiro et al. (2003)
**Proboscids**									
Asian elephant (*Elephas maximus)*	–	–	–	✘	–	–	–	–	Ketchaisri et al. (2019)
**Ungulates**									
Domestic horse (*Equus caballus)*	–	–	–	✘	–	✓	–	–	McKinley and Sambrook (2000), Proops and McComb (2010)
Domestic pig (*Sus scrofa domesticus)*	✘	–	–	✓	–	✓	–	–	Byrne et al. (2001), Nawroth et al. (2014)
Dwarf goat (*Capra aegagrus hircus)*	✓	–	–	✘	–	–	–	–	Kaminski et al. (2005), Nawroth et al. (2015), Schaffer et al. (2020)
Guanaco (*Lama guanicoe)*	✘	–	–	–	–	–	–	–	Schaffer et al. (2020)
Llama (*Lama glama)*	✓	–	–	–	–	–	–	–	Schaffer et al. (2020)
Mouflon (*Ovis orientalis orientalis)*	✓	–	–	–	–	–	–	–	Schaffer et al. (2020)
**PRIMATES**									
**Great apes**									
Bonobo (*Pan paniscus)*	✓	✓	–	✓	–	–	–	✓	Bräuer (2005, 2006), Bräuer et al. (2005), Okamoto-Barth et al. (2007), Mulcahy and Call (2009), Herrmann et al. (2010), Kano and Call (2014)
Chimpanzee (*Pan troglodytes)*	✓	✓	✓	✓	✓/✘	✓	✓	✓	Itakura (1996), Povinelli and Eddy (1996a,b, 1997), Call et al. (1998), Itakura and Tanaka (1998), Tomasello et al. (1998, 2001), Itakura et al. (1999), Hare et al. (2000, 2001), Karin-D'Arcy and Povinelli (2002), Okamoto et al. (2002, 2004), Bräuer et al. (2005), Melis et al. (2006), Okamoto-Barth et al. (2007), Mulcahy and Call (2009), Herrmann et al. (2010), Kano and Call (2014)
Gorilla (*Gorilla gorilla)*	✓	✓	–	✓/✘	–	–	–	✓	Peignot and Anderson (1999), Bräuer et al. (2005), Okamoto-Barth et al. (2007), Byrnit (2009), Schmid et al. (2017)
Orangutan (*Pongo pygmaeus)*	✓	✓	✓	✓/✘	–	✘	✘	✓	Itakura (1996), Itakura and Tanaka (1998), Kaplan and Rogers (2002), Byrnit (2004), Bräuer et al. (2005), Okamoto-Barth et al. (2007), Mulcahy and Call (2009), Gretscher et al. (2012), Kano and Call (2014)
**Gibbons**									
Hoolock gibbon *(Hoolock leuconedys)*	–	–	–	–	–	–	✓	–	Sánchez-Amaro et al. (2020)
White-handed gibbon (*Hylobates lar)*	✓	–	–	✓	–	–	–	✘	Inoue et al. (2004), Liebal and Kaminski (2012)
Pileated gibbon (*Hylobates pileatus)*	✓	–	–	–	–	–	–	✓/✘	Horton and Caldwell (2006), Liebal and Kaminski (2012)
Siamang (*Symphalangus syndactylus)*	✓	–	–	–	–	–	–	✘	Liebal and Kaminski (2012)
Silvery gibbon (*Hylobates moloch)*	✓	–	–	–	–	–	✓	✘	Liebal and Kaminski (2012), Sánchez-Amaro et al. (2020)
**Lemurs**									
Ruffed lemur (*Varecia veriegata rubra)*	✘	–	–	–	–	–	✘	–	Sandel et al. (2011)
Black lemur (Eul*emur macaco)*	✓/✘	–	✘	✓	–	–	✘	–	Itakura (1996), Anderson and Mitchell (1999), Ruiz et al. (2009), Sandel (2011)
Brown lemur (*Eulemur fulvus)*	✓	–	✘	✓	–	–	–	–	Itakura (1996), Ruiz et al. (2009)
Mongoose lemur (*Eulemur mongoz)*	✘	–	–	–	–	–	✘	–	Sandel et al. (2011)
Ringtailed lemur (*Lemur catta)*	✓	–	–	–	–	–	✓	–	Shepherd and Platt (2008), Sandel et al. (2011)
**New World monkeys**									
Common marmoset (*Callithrix jacchus)*	✘	–	–	✘	✓	–	✓	✘	Burkart and Heschl (2006), Burkart and Heschl (2007)
Cotton–top tamarin (*Saguinus oedipus oedipus)*	✓	–	–	✓	–	–	–	–	Santos and Hauser (1999), Neiworth et al. (2002)
Spider monkey (*Ateles geoffroyi)*	–	✓	–	–	–	–	–	✘	Amici et al. (2009)
Squirrel monkey (*Saimiri sciureus)*	✓	–	✘	–	–	–	–	–	Itakura (1996), Anderson et al. (2005)
**Old World monkeys**									
Barbary macaque (*Macaca sylvanus)*	✓	–	✓	–	–	–	–	–	Teufel et al. (2010), Rosati and Santos (2017)
Crested macaque (*Macaca nigra)*	✓	–	–	–	–	–	–	–	Micheletta and Waller (2012)
Long-tailed macaque (*Macaca fascicularis)*	✓	✘	–	–	✓	–	–	✓	Goossens et al. (2008, 2012), Overduin-de Vries et al. (2014)
Pig-tailed macaque (*Macaca nemestrina)*	✓	–	✘	–	–	–	–	–	Itakura (1996), Tomasello et al. (1998), Ferrari et al. (2000, 2008), Paukner et al. (2007)
Rhesus macaque (*Macaca mulatta)*	✓	✘	✘	✓/✘	–	–	✓	–	Anderson et al. (1996), Itakura (1996), Emery et al. (1997), Tomasello et al. (1998, 2001), Lorincz et al. (2000), Flombaum and Santos (2005), Shepherd et al. (2006), Roy et al. (2014), Rosati et al. (2016), Bettle and Rosati (2019)
Stump-tailed macaque (*Macaca arctoides)*	✓	–	✘	–	–	–	–	–	Itakura (1996), Tomasello et al. (1998), Anderson and Mitchell (1999)
Tonkean macaque (*Macaca tonkeana)*	–	–	✘	–	–	–	–	–	Itakura (1996)
Pigtail macaque (*Macaca nemestrina)*	✓	–	–	–	–	–	–	–	Ferrari et al. (2000, 2008), Paukner et al. (2007)
Capuchin monkey (*Cebus apella)*	✓	✓	✘	✘	✘	✓	–	✘	Anderson et al. (1995), Itakura (1996), Itakura and Anderson (1996), Vick and Anderson (2003), Hare et al. (2003), Anderson et al. (2005), Amici et al. (2009), Defolie (2015)
White-faced capuchin (*Cebus capucinus)*	–	–	✘	–	–	–	–	–	Itakura (1996)
Diana monkey (*Cercopithecus diana diana)*	✓	–	–	–	–	–	–	✓	Scerif et al. (2004)
François’ langur (*Trachypithecus francoisi)*	✓	–	–	–	–	–	–	–	Chen et al. (2017)
Golden snub-nosed monkey (*Rhinopithecus roxellana)*	✓	–	–	–	–	–	–	–	Chen et al. (2017)
Olive baboon (*Papio anubis)*	✓	–	–	–	–	–	✓	–	Parron and Meguerditchian (2016), Vick and Anderson (2003)
Sooty mangabey (*Cercocebus atys torquatus)*	✓	–	–	–	–	–	–	–	Tomasello et al. (1998)
**REPTILES**									
**Crocodylians**									
American alligator (*Alligator mississippiensis)*	✓	✘	–	–	–	–	–	–	Zeiträg et al. (2022)
**Lizards**									
Bearded dragon (*Pogona vitticeps)*	✓	✘	–	–	–	–	–	–	Siviter et al. (2017)
Leopard gecko (*Eublepharis macularius)*	✓	–	–	–	–	–	–	–	Simpson and O’Hara (2019)
**Tortoises**									
Red-footed tortoise (*Geochelone carbonaria)*	✓	–	–	–	–	–	–	–	Wilkinson et al. (2010b)
**FISHES**									
Archerfish *(Toxotes chatareus)*	✓	–	–	–	–	–	–	–	Leadner et al. (2021)

GFD = Gaze following into distance, GGF = Geometrical gaze following, GFBS = Gaze following behind subject, OC = Object choice using gaze cues, COC = Competitive object choice, FTC = Food theft competition, GKT = Guesser-knower task, CB = Checking back,

✓ = succeeded in this paradigm, ✘ = failed in this paradigm, ✓/✘ = conflicting findings, − = not tested in this paradigm.

Many different factors can influence a species’ performance in gaze following experiments. Realistically, it is difficult to take all of the following issues into account. Nevertheless, this summary should provoke researchers to closely familiarize themselves with the species and subjects they are testing. These considerations should further be taken into account when designing gaze following experiments.

### Experimental paradigms

#### Visual co-orientation

Visual co-orientation is commonly tested in two ways, in line with Povinelli and Eddy’s low-and high-level model of gaze following (Povinelli and Eddy, 1996a). According to this first account of gaze following in chimpanzees (*Pan troglodytes*), low-level gaze following is a simple co-orienting reflex that does not require any mentalistic attributions. High-level gaze following, on the other hand, requires a representation of others’ visual perspectives.

To test these models, two different experimental setups are used: gaze following into the distance (from here on GFD) and geometrical gaze following (from here on GGF). In the former, a demonstrator is gazing either up or to the side while the reaction of the observer is recorded. This is considered to require only low-level cognitive abilities without an attribution of mental states. GGF refers to tracking gazes around barriers. It is elicited by a demonstrator gazing toward a location behind a barrier that is not seen from the subject’s location. If an observer were to simply co-orient with the demonstrator, one would expect it to look at the barrier. An observer capable of GGF, though, moves around the barrier to inspect the target of the demonstrator’s gaze. This is regarded as high-level gaze following as it is believed to require an understanding of visual perspectives (Bräuer et al., 2005).

Most studies on gaze following in animals have focused on GFD. GGF has been tested in primates (Bräuer et al., 2005; Amici et al., 2009; Bettle and Rosati, 2019), canids (wolves: Range and Virányi, 2011; domestic dogs: Met et al., 2014), birds (Corvids: Bugnyar et al., 2004; Schloegl et al., 2007, 2008b; Northern bald ibises: Loretto et al., 2009; European starlings: Butler and Fernández-Juricic, 2014), and two reptile species (Bearded dragons: Siviter et al., 2017; American alligators: Zeiträg et al., 2022).

#### Object choice

In the object choice paradigm, an experimenter indicates the location of a hidden piece of food through a cue such as pointing, tapping, or gazing. In this review, we focus exclusively on gaze cues and their use in locating food. Several variations of the object choice paradigm exist: classic object choice, competitive object choice, food theft competition, and the guesser-knower task, which will be explained below.

##### Classic object choice

In the classic object choice paradigm, an experimenter is cueing the subject towards one of two objects that is baited with food. These objects are usually small containers such as cups. When the animal has chosen, the remaining object is removed from access of the subject.

In experiments where gazes are used as cues, a distinction is made between *gaze*—referring to a shift of both, head and eye direction—and *glance*—referring to a shift of the eyes alone (e.g., Neiworth et al., 2002; Scheumann and Call, 2004; Burkart and Heschl, 2006).

Surprisingly, most tested species struggled with this paradigm and failed to use gaze to locate food (Anderson et al., 1995; Schloegl et al., 2008a; Giret et al., 2009). The disparity between the seemingly ubiquitous gaze following, but poor results in object choice experiments, suggests a difference in the mental processing of gaze direction and its use in finding food.

Call et al. (2000) argued that there is a functional disparity between following gaze and foraging, and that chimpanzees do not seem to apply their elsewhere proven gaze following skills in foraging contexts. In the same study the authors found that vocalizations and other behaviors, such as approaching the baited object, significantly increased performance. The authors argued that these additional behaviors drew the subjects’ attention towards the demonstrator’s head direction which was then used to locate the hidden food. They called this the *attention boosting hypothesis.*

However, this hypothesis does not explain why many species follow gaze, as they seemingly cannot use it for locating food. Therefore, one must disentangle whether gaze following takes place in foraging contexts at all, or whether many species are unable to infer the location of food from observed gaze directions.

One study on common marmosets (*Callithrix jacchus*) showed that the monkeys followed the experimenter’s gaze to the correct container, but were unable to find the hidden food (Burkart and Heschl, 2007). Call et al. (2000) argued in their study that chimpanzees did not understand the communicative and informative intent of the human experimenter. That would also explain why adding vocalizations can enhance object choice performance, as it might convey and emphasize the communicative intent of the situation to the subject. Similarly, Hauser and Wood (2011) found improved performances of rhesus macaques (*Macaca mulatta*) in an object choice task when using “communicative gaze gestures”, i.e., wide open eyes and jerking the head several times towards the target object. However, Burkart and Heschl (2007) provide an alternative explanation for their marmosets’ behavior: looking at an object might be interpreted as an indication of ownership and lead to avoidance of that object. While this explanation seems reasonable for the cooperatively breeding marmosets, it does not explain the behavior of competitive species such as chimpanzees.

In fact, two lemur species, black lemurs (*Eulemur macaco*) and brown lemurs (*Eulemur fulvus*), have been reported to react contrary to common marmosets (Ruiz et al., 2009). In an object choice experiment, the lemurs were more likely to locate hidden food after they had successfully followed the gaze direction from a conspecific’s photograph. The poor performance of many species in object choice experiments is according to this study caused by low gaze following rates. The authors called this effect *gaze priming*, i.e., an increase of saliency of an object or location through following gaze direction. However, it should be noted that this is, to our knowledge, one of only two studies using conspecific demonstrators (even though just photos) in an object choice experiment, which might have significant impacts on subjects’ behaviors (for a study on ravens, see: Schloegl et al., 2008a).

Unfortunately, object choice studies rarely report whether subjects had followed the demonstrator’s gaze toward the correct object or not. More of these reports are needed to understand the reasons behind the poor performance of many species in the object choice paradigm. Nevertheless, two factors have been identified to improve subjects’ performance. Chimpanzees have been found to perform better when leaving the experimental area after each trial, and having to approach the experimenter at the beginning of each new trial (Barth et al., 2005). This indicates an attentional issue in “classic” object choice setups, where subjects stay in the same place between trials.

Secondly, chimpanzees perform better when the food is hidden in, or behind, an object that allows visual access to the experimenter, but blocks the subject’s view (Call et al., 1998). This is the case with, for example, tubes or barriers, where the experimenter can see the hidden food while cueing its location to the subject. It could be argued that animals do not perceive an experimenter as knowledgeable of the food’s location when they cannot see the hidden food at the time they are giving cues to the subject (for alternative explanations see Call et al., 1998).

Though many species struggle with object choice experiments, it seems as though changes to the setup can significantly improve subjects’ performances. For that reason, alternative versions of the classic object choice paradigm have been introduced.

##### Competitive object choice

One alternative explanation for the poor performance of many species in object choice experiments could be that informing others about the location of hidden foods does not come naturally to many animals. Several authors have argued that especially competitive species, such as chimpanzees, would hardly ever inform conspecifics about hidden food, making the experimental setup much less relevant to them. For that reason, Hare et al. (2000) invented the competitive object choice paradigm. This experiment has, to our knowledge, only been conducted on primates. In the competitive object choice setup, two individuals, one dominant and one subordinate, are observing an experimenter placing two food items in an experimental room. One of the food items is visible to both participants, while another is only visible to the subordinate. Once the food is placed, both individuals are released into the experimental room. If the subordinate understood the visual perspective of the dominant, it would be expected to first go and collect the hidden piece of food to avoid competition with the dominant. A variety of primate species appeared to demonstrate an understanding of others’ visual “perspectives” in the competitive object choice paradigm (see below).

In an additional condition in this paradigm, Hare et al. (2000) introduced a delay to the release of the dominant in relation to the subordinate in order to rule out that the choice made by the subordinate would be influenced by the approach and choice of the dominant competitor, i.e., that the subjects were simply choosing the food item that the dominant was *not* choosing.

Chimpanzees appeared to take visual access of the dominant into account by choosing the food item only visible to them over the one visible to both. Subordinates were still choosing the food item only visible to them when being released with a head start. The same behavior was found in common marmosets (Burkart and Heschl, 2007) and long-tailed macaques (*Macaca fascicularis*; Overduin-de Vries et al., 2014). Subordinate capuchin monkeys (*Cebus apella*) also displayed a preference for the hidden food (Hare et al., 2003). This preference, however, broke down when granting the subordinate a head start into the arena.

Using cross-correlations of subordinates’ and dominants’ behaviors, Hall et al. (2014) showed that subordinate chimpanzees rarely follow the dominant’s gaze in a competitive object choice setup. This supports the hypothesis that subordinates are not simply choosing based on the other’s behavior. Dominants, on the other hand, were following subordinates’ gazes and adapting their foraging technique to that of the conspecific with privileged knowledge about the location of food.

To further control for behavior-reading, Overduin-de Vries et al. (2014) introduced a one-way mirror in their study on long-tailed macaques that allowed the subordinate to see the dominant, creating the illusion of a competitive situation. The dominant, however, could not actually see the subordinate or the food, excluding the possibility of giving behavioral cues about which food they will be choosing to the subordinate. Interestingly, in this setup fast subordinates were often observed collecting both food items. In those instances, subjects were going for the visible food item first, before collecting the food only visible to them. Slower individuals more often only chose the food item that was not visible to their competitor.

##### Food theft competition

In the food theft competition, the subject’s choice of object is mediated by its gaze sensitivity in a competitive setting. Two food items are placed in the experimental room, with an experimenter monopolizing one of the items through closer proximity or visual orientation to the item. If the subject chooses the object by the experimenter, the object is immediately removed from access and the trial ends. If the subject chooses the object not contested by the experimenter, it may eat the food item.

A variety of primate species have been successful in this paradigm, including gibbons (Sánchez-Amaro et al., 2020), Old world monkeys (Vick and Anderson, 2003; Flombaum and Santos, 2005), and one species of lemur (ringtailed lemurs*, Lemur catta*), while three other lemur species failed in this study (mongoose lemurs, *Eulemur mongoz*; black lemurs, *Eulemur macaco*; ruffed lemurs,*Varecia variegata rubra*; Sandel et al., 2011).

In a more complex setup, chimpanzees could reach for a banana either through an opaque or a transparent tunnel (Melis et al., 2006). Chimpanzees preferred to conceal their actions through choosing the opaque tunnel. Orangutans did not show a preference in a replication of this experiment, failing to show an understanding of visual access (Gretscher et al., 2012).

##### Guesser-knower task

In this object choice paradigm, the subject’s understanding of a demonstrator’s visual access is tested. The standard setup involves a subject and two demonstrators. Food is then hidden outside the visual access of the subject, while one demonstrator has visual access to the baiting process (the knower), and the other demonstrator does not (the guesser). This can be achieved through the guesser being absent (e.g., Maginnity and Grace, 2014), turned away (e.g., Call et al., 2000), or having their eyes closed (Proops and McComb, 2010) during baiting. Both demonstrators (or one demonstrator in different trials) subsequently perform a cue towards one of two objects. A subject with an understanding of visual access should prefer the object indicated by the informed demonstrator.

Two primate species have been successful in this paradigm: chimpanzees (Call et al., 2000) and capuchin monkeys (Defolie et al., 2015). Moreover, some domesticated species could infer visual access of humans: domestic dogs (*Canis familiaris*; Virányi et al., 2004; Maginnity and Grace, 2014; Catala et al., 2017), domestic horses (*Equus caballus*; Proops and McComb, 2010; Ringhofer et al., 2021), and domestic pigs (*Sus scrofa domesticus*; Byrne et al., 2001).

Only one bird species has been subject to the guesser-knower task: common ravens (*Corvus corax*; Bugnyar, 2011). Ravens were watching a human experimenter cache food in the presence of two conspecifics: a guesser, whose view was obstructed during caching, and a knower, that could witness the caching process. After releasing the subject and either of the two conspecifics into the arena, subjects were pilfering the caches quicker when in competition with an informed conspecific, showing that they were taking others’ visual access into account.

### Demonstrators

The experimental study of gaze following requires a gaze cue by a demonstrator. Such a demonstrator should on command gaze towards a specific location. To ensure controlled testing conditions, the majority of studies, especially on primates—have used human demonstrators (for an overview see: Rosati and Hare, 2009). This allows for controlling parameters such as looking time, specific target of gaze and the disentanglement of head direction and eye-gaze alone. While it is doubtless beneficial to keep testing conditions as controlled as possible, the relevance of human gaze cues to animals, especially non-primates, has been debated.

The large body of literature on gaze following in a variety of species indicates that many animals can indeed follow the gaze of a human demonstrator—even around barriers. That, however, does not mean that the frequency and sophistication are representative of the species’ socio-cognitive potential. Though commonly brought up in discussions, few studies have directly addressed this topic.

One study on cotton-top tamarins (*Saguinus oedipus oedipus*) for example showed that the monkeys did not follow the gaze of humans, but only of conspecifics (Neiworth et al., 2002). Contrarily, other primate studies have reported comparable responses to human and conspecific demonstrators (Tomasello et al., 1998; Hare and Tomasello, 2004; Herrmann et al., 2007). However, Byrnit (2004) described that non-enculturated orangutans, i.e., parent-raised, failed to use human gaze cues to identify a target object. Similarly, only enculturated chimpanzees have been found to be sensitive to visual attentive states of human experimenters in a guesser-knower task (Call et al., 2000).

If previous exposure to humans indeed impacts animals’ sensitivity to human gaze cues, the ability to follow human’s gaze might vary significantly depending on age and experience. To our knowledge, only one observational account of such a developmental effect exists from common ravens (Schloegl et al., 2007). The authors observed the first spontaneous co-orientations of raven nestlings to their conspecifics approximately 7 weeks before they started to react to the gaze of a human experimenter.

Previous exposure to humans could also explain the excellent performance of domestic dogs using human gaze cues in object choice tasks (e.g., Miklósi et al., 1998; Hare and Tomasello, 1999; Agnetta et al., 2000; Soproni et al., 2001). It has been argued that this is the result of their long history of domestication and exposure to humans. A direct comparison of dogs and chimpanzees in an object choice task revealed that dogs indeed outperform chimpanzees when using communicative cues given by human experimenters, such as pointing and gazing. Chimpanzees, on the other hand, were better at inferring the location of the hidden food from causal cues such as the sound produced when shaking the baited container or the shape of a board that is slanted because of food being hidden under it (Bräuer et al., 2006). This further supports the hypothesis that the poor performance of chimpanzees in object choice tasks is not caused by a lack of understanding, but rather because they do not recognize the communicative intent of the human experimenter and therefore fail to use it as a cue.

Hattori et al. (2010) compared the responses of humans and chimpanzees to con-and allospecific gaze cues and found that chimpanzees follow human gaze significantly less compared to conspecific gaze and moreover look longer at faces of other chimpanzees than of humans. Interestingly, humans were equally sensitive to gazes of both demonstrator species.

Taken together, these findings indicate that some enculturated individuals and domesticated species might respond stronger to human compared to conspecific gaze cues. However, a recent study on gaze following in domesticated ungulates found that even domesticated species preferred to follow conspecific gazes (Schaffer et al., 2020). This suggests that for the majority of species, the use of conspecific demonstrators would be beneficial and that many studies might not have revealed the full gaze following potential of their tested species.

To keep experimental conditions controlled while using conspecific demonstrators, some studies have used photographs and even videos of conspecifics to induce gaze following responses. This seems to work surprisingly well. Primates such as rhesus macaques (Lorincz et al., 2000), Diana monkeys (*Cercopithecus diana diana*; Scerif et al., 2004), and two lemur species (Ruiz et al., 2009) followed the gaze direction of a photograph of a conspecific to an object. Several primate species have also been found to co-orient to videos of conspecifics, such as chimpanzees, bonobos, orangutans (Kano and Call, 2014) and rhesus macaques (Emery et al., 1997).

However, not only primates have been found to co-orient with artificial stimuli. Bearded dragons (*Pogona vitticeps*) followed the video of a conspecific gazing up and to the side (Siviter et al., 2017), and even archerfish co-oriented themselves with a photograph of a conspecific (Leadner et al., 2021). Butler and Fernández-Juricic (2014) have gone as far as creating a robot version of a European starling (*Sturnus vulgaris*) to test for GGF skills in this species. The subjects followed the robot’s gaze behind barriers, suggesting that artificial demonstrators can be used in GGF studies.

### Eye morphology

One problem when interpreting gaze following is knowing exactly where an animal is looking. Birds, for example, can switch from lateral to binocular vision, therefore the target of their gaze cannot be deduced from their eye orientation (Dawkins, 2002). In animal studies, the target of gaze can only be inferred. This is usually done by extrapolating postural indicators such as beak or snout direction.

Similarly, when using conspecifics as demonstrators, they usually orient their bodies or at least the head toward that location. In the presence of a co-orienting response, it is therefore difficult to discern which cue the observer has been following, the direction of the body, the head, or the eyes alone. Some studies on primates have started to disentangle these cues, but again with human demonstrators to specifically control the body parts orienting toward the target (e.g., Anderson et al., 1996; Neiworth et al., 2002; Burkart and Heschl, 2006).

Differences in the accuracy of gaze tracking might be caused by varying eye morphologies. It has long been believed that human eyes are unique in their salience through the contrast between the white sclera and the dark central iris. This eye morphology has been thought to allow for accurate identification of the target of the conspecifics’ gaze, and has been an explanation for the ability of humans to use eye gaze alone, in comparison to non-human primates that rely on head direction to track gaze (Tomasello et al., 2007). This has been called the *cooperative eye hypothesis*. However, a recent study found that despite the differences in scleral pigmentation, the contrast between sclera and iris is comparable for great apes and humans (Perea-García et al., 2019). Thus, the contrast of the eyes alone is not sufficient to explain the differences in the sophistication of gaze following between humans and non-human primates.

Kano et al. (2022) recently shed new light on this question. Humans and chimpanzees were asked to choose one out of three pictures of faces of humans and chimpanzees. The task was to pick the face with averted eyes. In different conditions, parameters of the eyes, such as brightness and size were manipulated. Humans and chimpanzees were both better at discriminating the human’s eye direction and this effect was most prevalent in visually challenging conditions, i.e., when the pictures were shaded or small. The authors argued that the uniformly white sclera of humans, rather than the contrast of sclera and iris, is responsible for human gaze following skills as it makes it easier to follow eye movement alone rather than head direction.

Other animals, however, do not possess such an eye morphology and need to rely on other directional cues, such as head directions. While it seems likely that the evolution of a conspicuous eye morphology in humans has refined the ability to extract social information from the eyes alone, it does not mean that other animals lacking this morphology cannot follow others’ gaze directions. They, however, need to rely on different directional cues. In ravens, for example, the direction that the beak is pointing towards is a clearer indicator of others’ visual attention than a shift of the small, dark, and evenly colored eyes.

The phylogenetic ubiquity of co-orientations in combination with evidence for an evolutionary conserved subcortical pathway guiding gaze following responses moreover indicate that the evolutionary roots of this skill run deeper than the evolution of human eye morphologies. It is, hence, more likely that uniquely human abilities’ in extracting directional cues from shifts of eye directions alone are a species-specific extension of the gaze following capacities of other animals rather than a separate, more sophisticated way of extracting directional information.

### Emotions

Another factor influencing gaze following are the emotions associated with attentional shifts. Goossens et al. (2008) have studied this effect in long-tailed macaques through a human experimenter accompanying their gaze shifts with mimicked facial expressions representing different emotional states for the monkeys, namely aggression, submission and affiliation. The authors reported that long-tailed macaques were more likely to follow gaze when the attentional shift was accompanied by facial expressions of fear and submission. This could be an indication that gaze following can be employed flexibly in socially meaningful situations.

Teufel et al. (2010) found that facial expressions generally facilitated gaze following in Barbary macaques (*Macaca sylvanus*), resulting in a higher gaze following frequency in trials where the gaze cue was accompanied by a facial expression. This effect was present in all age classes but was reduced in older individuals, indicating an impact of experience on modulating gaze following responses. While it might be beneficial for developing individuals to pay attention to as much detail in their social and physical environment as possible, more experienced individuals might only attend to events that are critical to them. In this study, the strongest effect on gaze following was observed when gaze cues were accompanied by “commenting” facial expressions. These are facial expressions that Barbary macaques make when observing third-party interactions. The authors therefore argued that the augmenting effect of facial expressions on gaze following responses might have evolved to facilitate the acquisition of social knowledge through drawing attention to social interactions. In humans, negative facial expressions, such as disgust and fear, have been shown to cause quicker gaze following responses compared to happy or neutral faces (e.g., Pecchinenda et al., 2008; Matsunaka and Hiraki, 2014). In contrast, a similar study on pigtail macaques (*Macaca nemestrina*) did not find evidence for an effect of facial expressions on gaze following performances (Paukner et al., 2007).

These experimental setups are limited firstly to emotions that are visible in facial expressions, and secondly to species exhibiting facial expressions, which is mainly the case in primates. This however does not mean that other animals are not affected by observing facial expressions. Horses have, for example, been shown to follow human gaze less frequently when the experimenter expressed disgust, indicating interspecies interpretation of facial expressions (Baba et al., 2019).

Not only does the emotional state of the demonstrator impact gaze following performances, but also the emotional state of the subject. Putnam et al. (2016), for example, found that rhesus macaques were more likely to follow gaze in response to videos of a conspecific after inhaling intranasal oxytocin. The oxytocin might enhance the motivation for social interactions through receptors projecting onto social cortical regions, such as the STS.

### Social dynamics

Social dynamics, i.e., the relationship between the demonstrator and the observer, can affect the likelihood of gaze following. For example, in rhesus macaques, social status impacts gaze following (Shepherd et al., 2006). While low-status males followed the gaze of all familiar conspecifics, high-status males exclusively followed the gaze of other high-status individuals. For low-ranking individuals, it might be crucial to monitor the behavior of their conspecifics to avoid aggressive encounters, while high-ranking individuals are only threatened by other high-ranking monkeys. This shows that gaze following responses may be modulated by social context to optimize gathering of relevant social information. Contrarily, in Barabary macaques, the social status of the demonstrator does not influence gaze following responses (Teufel et al., 2010).

But status, or even kinship, is not always the most important social factor in gaze following. In crested macaques (*Macaca nigra*), such factors had no influence, but instead strong positive bonds between individuals facilitated quicker responses in gaze following (Micheletta and Waller, 2012). The authors argued that social information from friends might be more relevant due to shared interests and motivations. Also, when locating resources such as food, the competition might be reduced between affiliates. A different motivation to follow a friend’s gaze could be to monitor social interactions involving affiliates to support in conflicts or provide post-conflict affiliation.

Social dynamics also play a role when using human demonstrators. This effect was reported in a study on object choice in jackdaws (von Bayern and Emery, 2009). The birds only responded to gaze cues by a familiar experimenter. Similar to the crested macaques, familiarity could make it easier for jackdaws to predict the other’s actions, especially in a competitive situation.

### Sex differences

Lastly, an effect that has rarely been tested in animals, is the impact of sex on gaze following. In humans, women show a stronger gaze cueing effect, i.e., they are more likely to follow other’s gaze (Bayliss et al., 2005). They moreover react quicker to gaze than men. Interestingly, this sex-difference was correlated with self-reported empathy levels (Alwall et al., 2010).

The only reports of such an effect in animals stem from rhesus and Barbary macaques. Both studies found that females were more likely to follow gaze (Paukner et al., 2007; Rosati et al., 2016). In contrast, a different study on Barbary macaques found that sex had no effect on gaze following (Teufel et al., 2010). More studies are needed to understand sex differences in gaze following and their connections to empathy in animals.

## Gaze following in mammals

Primates play a central role in the study of gaze following as most experimental methods have been developed in primate studies (see sections above), with chimpanzees being the first animal species to be tested in this paradigm. Here, we provide a brief overview of the current state of knowledge on gaze following in primates (for more detailed reviews see: Emery, 2000; Zuberbühler, 2008; Rosati and Hare, 2009; Shepherd, 2010).

To date, reports of GFD of at least some members from all major primate radiation exist: apes, including great apes (e.g., Bräuer et al., 2005) and gibbons (Horton and Caldwell, 2006), Old World monkeys (e.g., Emery et al., 1997), New World monkeys (e.g., Neiworth et al., 2002), and lemurs (e.g., Shepherd and Platt, 2008; but see Ruiz et al., 2009).

The number of GGF studies is lower. Evidence for gaze following around barriers exists from all major primate radiations besides the lemurs: great apes (e.g., Bräuer et al., 2005), Old World monkeys (e.g., Goossens et al., 2012) and one species of New World monkey (common marmosets: Burkart and Heschl, 2006).

Many primates have been found to struggle with using gaze cues to find food in the classic object choice task (e.g., Anderson et al., 1995; Neiworth et al., 2002; Burkart and Heschl, 2006). It seems unlikely that primates are incapable of locating food based on experimental cues. It rather appears that they fail to understand the communicative intent of a human experimenter. A competitive version of this experiment improved the performance of some species in that they avoided food items that dominant conspecifics were looking at (chimpanzees: Hare et al., 2000; capuchin monkeys: Hare et al., 2003; common marmosets: Burkart and Heschl, 2007; long-tailed macaques: Overduin-de Vries et al., 2014). Successful performances in this task are usually interpreted as an understanding of visual access in the tested species, though close attention needs to be paid to exclude the possibility of solving the task through behavior-reading.

Few more primate species have been tested in other variations of the object choice paradigm: food theft competition and guesser-knower task. Many primates seem successful in a food theft competition task (olive baboons, *Papio anubis*: Vick and Anderson, 2003; rhesus macaques: Flombaum and Santos, 2005; chimpanzees: Melis et al., 2006; ringtailed lemurs: Sandel et al., 2011; gibbons: Sánchez-Amaro et al., 2020), which supports the hypothesis that a competitive setting is more relevant to a variety of species. Two species have moreover been found to infer visual access of human experimenters in a guesser-knower task, namely chimpanzees (Call et al., 2000) and capuchin monkeys (Defolie et al., 2015).

Other than primates, the largest body of literature on the use of gaze cues in mammals stems from domestic dogs. Dogs successfully use variations of gaze to locate hidden food, making them significantly better at this task than any tested primate species (Hare et al., 1998; Miklósi et al., 1998; Agnetta et al., 2000; McKinley and Sambrook, 2000; Soproni et al., 2001; Bräuer et al., 2006). The cues in these studies include gazing, gaze alternations, glancing, different numbers of objects to choose from, and different distances between experimenter and object, i.e., the cue being performed by an experimenter standing close to or far away from the baited container. In a guesser-knower task, dogs demonstrated an understanding of visual access by preferring the cue of the knower over the one of the guesser (Maginnity and Grace, 2014; Catala et al., 2017) and by begging more from a human facing the dog compared to one with their back turned (Virányi et al., 2004).

Interestingly, the results are less clear when it comes to GFD. It has been reported that dogs do not spontaneously follow human gaze into distant space (Agnetta et al., 2000). Their performance improved, though, when the target of gaze was clearly defined, or when the communicative intent was made clear through ostensive cues, such as calling the dogs’ name (Téglás et al., 2012; Werhahn et al., 2016; Duranton et al., 2017). In a comparison with pack-living domestic dogs and wolves (*Canis lupus*), wolves actually followed human gaze more frequently, while both species followed their packmates’ gaze at comparable rates (Werhahn et al., 2016).

An explanation for this phenomenon could be that dogs are overly focused on humans and struggle with directing their attention away from them to a location in the environment. Wallis et al. (2015), for example, reported that even a short training of dogs to seek eye contact with humans disrupted their gaze following responses. With respect to GGF, dogs seemed capable of tracking humans’ gazes around visual barriers, and even more so in a foraging context, i.e., when they were aware of food being hidden (Met et al., 2014). This suggests that domestication might favor the use of gaze cues to locate food but might simultaneously hinder spontaneous gaze following responses.

Other canids tested are wolves, dingoes (*Canis lupus dingo*) and silver foxes (*Vulpes vulpes*). Hand-raised wolves followed both, human and conspecific gaze into the distance and around barriers. They were found to follow humans’ gaze into distant space at 14 weeks, while GGF only developed after 6 months (Range and Virányi, 2011). The difference in ontogeny supports the hypothesis of two different cognitive processes developing at different times. The comparably later onset of GGF indicates a more complex underlying mechanism that takes longer to develop. Dingoes failed to locate hidden food using gaze cues given by a human experimenter (Smith and Litchfield, 2010). Domesticated silver fox kits performed comparable to dogs in an object choice tests with gaze cues and significantly better than their feral counterparts (Hare et al., 2005). Only one study on domestic cats (*Felis silvestris catus*) in an object choice paradigm exists. Cats were able to use human gaze to locate hidden food even without ostensive cues (Pongrácz et al., 2019). These studies support the hypothesis that previous exposure to humans and domestication drastically improve animals’ interpretation of experimenter-given cues.

A variety of farm animals have been subject to gaze following studies. Visual co-orientation has been tested in a number of ungulates: domestic goats (*Capra hircus*), llamas (*Lama glama*), guanacos (*Lama guanicoe*), and mouflons *(Ovis orientalis orientalis*; Kaminski et al., 2005; Schaffer et al., 2020). All these species, except for the guanacos, followed humans’ gazes into distant space. In an object choice task, however, goats failed to use experimenter-given gaze cues to find hidden food (Kaminski et al., 2005). In a follow-up study, Nawroth et al. (2015) showed that dwarf goats (*Capra aegagrus hircus*) have an understanding of visual attention of humans through increased anticipatory behaviors when a human was facing them compared to when they were not. However, they did not—just as many primate species—apply these skills in an object choice task.

In contrast, juvenile domestic pigs could find hidden food using a human’s head and body orientation, but interestingly did not follow head direction into distant space (Nawroth et al., 2014). In a guesser-knower task, pigs could choose to follow an informed or uninformed conspecific into one of two corridors, of which one has been baited with food before (Byrne et al., 2001). One pig successfully solved this task. These findings indicate that negative results from one testing paradigm do not necessarily predict a species’ performance in another paradigm.

Domestic horses identified a visually attentive experimenter over an inattentive one to approach for food, using body and head orientation as well as open or closed eyes as cues for visual attention. However, when the attentional cues were mixed, the horses’ performance broke down (Proops and McComb, 2010). They moreover passed a guesser-knower task and thus demonstrated sensitivity to visual attention of human experimenters (Ringhofer et al., 2021). In an object choice task, however, horses could not use alternating gaze as a cue to find hidden food (Proops et al., 2010).

Finally, some other mammalian species have been tested in object choice experiments. Asian elephants (*Elephas maximus*) did not use any human-given cues to locate food (Ketchaisri et al., 2019). Bottlenose dolphins (*Tursiops truncatus*) were able to use static and dynamic gaze to identify the correct object but failed with eye gaze alone (Tschudin et al., 2001; Pack and Herman, 2004). South African fur seals (*Arctocephalus pusillus*) used human gaze with head direction to identify the correct location of food (Scheumann and Call, 2004), while Gray seals (*Halichoerus grypus*) failed to do so (Shapiro et al., 2003).

The above-presented studies show a large variety in gaze following and the use of gaze in locating food within mammals. The majority of studies have focused on object choice paradigms, which appears inherently difficult to many animals. Gaze following studies in mammals are rare, limiting inferences of the status of this socio-cognitive skill within the mammalian clade.

## Gaze following in birds

After mammals, birds have been subject to most gaze following studies, especially corvids. This group is generally regarded among the most cognitively complex birds. Within the corvids, the common raven has been most extensively studied. Ravens follow the gaze of a human experimenter into distant space and geometrically around barriers (Bugnyar et al., 2004). While ravens already followed the experimenter’s look-ups as fledglings, GGF occurred only after 6 months. The same developmental trajectory was found in another corvid species, the rook (*Corvus frugilegus*; Schloegl et al., 2008b).

Both studies provided similar explanations for the development of gaze following skills, namely different ecological valences of the two modes of gaze following. Scanning the sky might serve as anti-predatory response and would therefore be a crucial cue already for fledglings. Looking around barriers, on the other hand, might serve as a cue to food sources, which is not important to fledglings due to parental care. Moreover, the emergence of GGF coincides with the time when ravens first start hiding behind barriers to conceal their caching (Bugnyar et al., 2007), indicating a developmental milestone in the understanding of visual perspectives.

The same developmental pattern has been found in primates and other mammals, where GFD emerges early in the development (e.g., rhesus macaques: Ferrari et al., 2000; chimpanzees: Tomasello et al., 2001; wolves: Range and Virányi, 2011), and GGF develops significantly later (e.g., human infants: Scaife and Bruner, 1975; chimpanzees: Okamoto et al., 2004; wolves: Range and Virányi, 2011). A more likely explanation, thus, is that the two modes of gaze following require different cognitive processes that develop at different times. The gap in the development of the two modes indicates more complex cognitive processes involved in GGF that the early developing brain is not yet capable of. Gaze following skills appear to develop at comparable rates and in the same pattern in birds and mammals, even though their brain morphologies differ drastically.

Ravens were moreover found to habituate quickly to look-ups, but not to geometrical visual cues of an experimenter (Bugnyar et al., 2004; Schloegl et al., 2007). To solve the habituation problem, Schloegl et al. (2007) introduced a new experimenter when the ravens stopped reacting to gaze cues of the familiar experimenter. This increased gaze following responses, though the subjects did not respond as strongly as in initial demonstrations. This increase, however, subsided quickly, indicating a rapid generalization between experimenters. The authors explained the lack of habituation in the geometrical experiment by the natural tendency of ravens to cache food. When tracking gaze behind a barrier and not finding an interesting object there, ravens might expect the object to be hidden and that a continuous search could be advantageous. As a comparison, chimpanzees do not habituate to gaze cues without an interesting target until adulthood (Tomasello et al., 2001).

Ravens have also been tested in object choice. Schloegl et al. (2008a) investigated whether ravens can locate a hidden piece of food through a variety of experimenter-given cues. The ravens did not seem to use gaze cues, not even when the experimenter was kneeling closely to the target object while gazing at it. Interestingly, the ravens also did not respond to a conspecific giving gazing cues towards one of two locations. The authors argued that the functional value of GGF is to use visual barriers to cache food outside of view from competitors rather than to locate the caches of conspecifics. While this seems to be a reasonable explanation for the evolution of GGF in ravens, it does not explain its presence in other non-caching birds and mammals.

In a comparative study on caching rooks and non-caching jackdaws (*Corvus monedula*; Schloegl et al., 2008b), only the rooks followed the gaze of a human experimenter into distant space as well as geometrically. The authors found only weak evidence for gaze following in jackdaws even when using a conspecific demonstrator perhaps due to a higher vigilance in these birds that makes the detection of gaze follows very difficult.

In an object-choice situation, the jackdaws identified the correct food location using cross-distal pointing and alternating gazes of their caretaker. They did not respond to static cues such as static gaze or head direction and did not respond to cues from an unfamiliar human (von Bayern and Emery, 2009). The authors argued that the dynamic nature of the used cues—in contrast to the static cues—conveyed the communicative intent of the gaze. A similar effect has been found in primates when accompanying experimenter-given cues with gestures and behaviors stressing the communicative intent (Call et al., 2000; Hauser and Wood, 2011). Furthermore, these findings suggest that the negative results on gaze following in jackdaws in the above-mentioned study (Schloegl et al., 2008b) were likely due to methodological artefacts.

The ability to use experimenter-given cues in an object-choice task of a fourth species of the corvid family—Clark’s nutcracker (*Nucifraga columbiana*)—has been examined (Tornick et al., 2011). These birds are non-social, in contrast to the other tested corvid species. Most subjects immediately used a touch gesture to identify the location of hidden food, which can be explained by local enhancement. Additionally, the birds successfully learned to use point and gaze cues. The gaze cue consisted of both head and eye direction and was dynamic, i.e., the gaze was alternated between the subject and the goal location. Despite methodological differences in studies, it seems that Clark’s nutcrackers perform comparably to social corvid species. This indicates that the social-nonsocial dichotomy is not sufficient to explain the presence of socio-cognitive skills, as they might either be derived from a social ancestor, or might be advantageous without social living (for a more detailed discussion see: Wilkinson et al., 2010a).

Outside of the corvid family, GFD with a conspecific demonstrator has been found in Greylag geese (*Anser anser*; Kehmeier et al., 2011), African penguins (*Spheniscus demersus*; Nawroth et al., 2017) and Northern bald ibises (*Geronticus eremita*; Loretto et al., 2009). Only three non-corvid bird species have been tested in GGF. European starlings (Butler and Fernández-Juricic, 2014) and red junglefowl (*Gallus gallus*; Zeiträg et al., 2022) successfully tracked the gaze of a conspecific around a barrier, while Northern bald ibises (Loretto et al., 2009) failed to follow a conspecific’s gaze geometrically. However, due to the many positive accounts of GGF in other bird species, it is possible that this negative account of GGF is caused by experimental artefacts.

A recent study (Zeiträg et al., 2022) reported the first accounts of gaze following in palaeognath birds. These birds are the less neurocognitively derived of the two major bird clades—palaeognaths and neognaths. They have retained many ancestral features from the non-avian dinosaurs (for a more detailed discussion see below). In this study, the authors found that three palaeognath species—greater rheas (*Rhea americana*), emus (*Dromaius novaehollandiae*), and elegant crested tinamous (*Eudromia elegans*) were capable of GFD– both up and to the side—as well as GGF.

The only non-corvid bird species tested in an object-choice experiment is the African gray parrot (*Psittacus erithacus*; Giret et al., 2009). The experimenter-given cues in this study included different pointing cues and distal and proximal gaze cues. Only one parrot spontaneously used a combination of proximal sustained pointing and gazing, a second one was able to learn to use the same gesture. Gaze cues alone were insufficient for any subject to locate the food.

## Gaze following in reptiles

There are very few studies on gaze following in reptiles, likely because they are considered non-social and thus unsuitable subjects to study social cognition. However, two studies have shown that non-social reptile species have socio-cognitive skills such as social learning (Wilkinson et al., 2010a; Kis et al., 2015). Studying reptiles is crucial for understanding the evolution of social cognition in Sauropsida, the clade containing reptiles and birds. Many studies have focused on the cognitive skills of mammals and birds. However, when trying to make inferences about the emergence of cognitive traits—in particular in birds, data from reptiles is needed (Matsubara et al., 2017).

Wilkinson et al. (2010b) conducted the first study on gaze following in a reptile—the red-footed tortoise (*Geochelone carbonaria*). They showed that this species co-orients with a conspecific’s upward gaze. Since then, three more reptilian species have been found capable of GFD: bearded dragons (*Pogona vitticeps*; Siviter et al., 2017), leopard geckos *(Eublepharis macularius*; Simpson and O’Hara, 2019), and American alligators (*Alligator mississippiensis*; Zeiträg et al., 2022).

Only two of these studies have additionally investigated GGF. Evidence for GGF was neither found in bearded dragons (Siviter et al., 2017) nor American alligators (Zeiträg et al., 2022). Though only few studies on reptiles exist, the large phylogenetic difference between the tested species indicates that GFD is present in distantly related reptilian radiations. The absence of GGF could be a result of the limited number of studies, or of an actual absence of this skill in reptiles. The brains of mammals and birds have, compared to reptiles, substantially more neurons in their telencephalon and cerebellum—regions commonly associated with higher cognitive capacities (Kverková et al., 2022). This neuroanatomical difference could explain the absence of GGF in reptiles. However, more studies are needed to verify the absence of this skill in reptiles.

## Gaze following in other species

To understand the evolutionary roots of gaze following, data from distantly related animal taxa capable of using gazes of others is needed. However, several taxa are either understudied or have not been investigated at all. No studies on amphibians exist, and research on reptilians has just started to gain more attention.

One recent study investigated the use of attentional cues in archerfish (Leadner et al., 2021). These fish spit water jets at insects above the surface. The subjects in the study were trained to spit water at a target on a computer monitor above their tank. In the test, the fish were confronted with the picture of a conspecific on the screen, oriented toward the right or left. After that, a target appeared on the left or right of the screen, half of the time congruent with the side indicated by the fish on screen. Archerfish were quicker to spit water at the target when it aligned with the demonstrator’s orientation. However, fish cannot turn their heads independently of their body. Therefore, the cue was a full-body orientation, so it is not possible to discern which part of the cue conveyed the direction of attention.

Interestingly, the authors reported an absence of *inhibition of return* (IOR) in archerfish. IOR describes the inhibition of returning attention toward a location that has already been observed after shifting attention elsewhere as a result of peripheral cues (McKee et al., 2007). When following gaze cues though, the IOR is absent in humans as well as the studied archerfish. The authors argued that archerfish, just like humans, might possess neural substrates specialized in processing social cues.

Whether the described co-orienting behavior is a special adaptation of archerfish and their hunting style, or a skill shared among fish species in unknown. As described above, all vertebrates share an evolutionary old subcortical pathway that mediates fast, reflexive shifts in visual attention. More studies on fish and amphibian species are needed to verify whether the presence of this pathway is sufficient for the presence of gaze following in all vertebrates.

## The use of social information conveyed through gaze: Social predictions

What animals actually understand about the gaze of others has been debated since Povinelli and Eddy (1996a) first introduced the low-and high-level explanation of gaze following (see above). While GGF can be interpreted as an understanding of the referential nature of gaze, very few studies have looked closer into social predictions that animals form based on observed gaze.

In this context, *checking back* (also called *double looks*) is of special interest. Checking back was first described when human children were found to look back to an experimenter in the absence of a target in their line of sight (e.g., Scaife and Bruner, 1975; Butterworth and Cochran, 1980). Children start this gaze alternation at 8 months, comparably late in contrast to the early onset of visual co-orientation at 2 months (Scaife and Bruner, 1975). Developmental psychologists have interpreted this behavior as a sign of an understanding of the mental aspects of gaze, i.e., that gazing refers to a target in the environment. Through alternating gazes between the gazer and the location they have oriented their gaze towards, infants try to identify the correct gaze target. It has been reported that babies point at a target and then turn back to the gazer as if to confirm its correctness (Butterworth and Cochran, 1980).

In animals, this behavior has first been described in chimpanzees by Call et al. (1998) as the animal looking back to the experimenter in the absence of interesting objects in their line of sight. Since the first description of checking back in chimpanzees, it has been reported in other great ape species like bonobos (*Pan paniscus*), gorillas (*Gorilla gorilla*) and orangutans (*Pongo pygmaeus*; Bräuer et al., 2005; Okamoto-Barth et al., 2007), pileated gibbons (*Hylobates pileatus*; Horton and Caldwell, 2006) and Old World monkeys—Diana monkeys (Scerif et al., 2004), and long-tailed macaques (Goossens et al., 2008). Interestingly, though specifically studied, no evidence for checking back was found in two species of New World monkey—spider monkeys (*Ateles geoffroyi*) and capuchin monkeys (Amici et al., 2009).

Bräuer et al. (2005) found a comparable ontogenetic trajectory of checking back in non-human primates and human infants. All five species of great ape checked back at comparable rates, but age had a significant effect on the number of checking back instances. The behavior was absent in infants, was first observed in juveniles, and occurred most often in adults.

In line with the hypothesis that checking back shows an expectancy violation when the demonstrator’s gaze is not referring to a target in the environment, pileated gibbons were found to check back more when a target appeared in a location that was incongruent with the location indicated through the gaze direction of a human experimenter or the photograph of a conspecific (Horton and Caldwell, 2006). The same was found in Diana monkeys (Scerif et al., 2004). Long-tailed macaques checked back more often in gaze shifts accompanied by social facial expressions, indicating an overall heightened attention in socially relevant situations (Goossens et al., 2008).

However, the mentalistic interpretation of checking back in infants has received criticism. Corkum and Moore (1995) for example argued that young children only look back at adults to confirm their attention or because they have expectations of the gazer’s behavior in the current situation. In an experimental set-up such an expectation could be that the experimenter will orient their gaze towards a new location after a brief break. Looking back at the experimenter could therefore be a sign of expecting a new gaze cue.

Call et al. (1998) argued in their study on chimpanzees that their subjects might have just returned to their neutral forward orientation. Finding the experimenter still gazing towards a location might have triggered a second, independent co-orientation. However, Bräuer et al. (2005) ruled this alternative explanation out by observing that checking back increased with age, indicating a learning process over time, from a simple co-orienting reflex in infants and juveniles to a perspective-taking model in adults.

To experimentally test the function of checking back, Okamoto-Barth et al. (2007) investigated great apes’ checking back behaviors in a “meaningful” and a “meaningless” condition. In both conditions, the experimenter was looking towards a target. In the meaningful condition, the experimenter’s line of sight was blocked by an opaque barrier. As there was nothing of interest to be seen when following the experimenter’s gaze, the authors hypothesized that apes will be more likely to look back at the experimenter in this condition. In the meaningless condition, the barrier between experimenter and target had a window, so that the experimenter and the subject could see the target. In this condition, the authors expected less checking back behavior as following the experimenter’s gaze would lead the apes to discover the target. The hypotheses were confirmed, as the chimpanzees and bonobos checked back more often in the meaningful condition. Orangutans and gorillas on the other hand seemed insensitive to the differences in the barrier conditions, producing checking back behaviors in both. This insensitivity indicates that the occurrence of checking back alone might not be sufficient to show understanding of visual perspectives and the referential nature of gaze.

Perhaps surprisingly, a recent study discovered checking back in three species of palaeognath and one species of neognath birds (Zeiträg et al., 2022). This was the first-ever description of checking back in any bird species, while no such behavior was found in alligators. The discrepancy between the two is likely caused by differences in their neuroanatomy. Birds have significantly more neurons in their brains than crocodylians and non-avian reptiles in general. However, proportionally, the biggest increase of neuronal numbers is accounted for by the cerebellum (Kverková et al., 2022). The higher neuronal numbers in the cerebellum of birds could explain the presence of checking back behaviors as this structure is believed to be involved in the formation of so-called *internal forward models*. These models are top-down processes using prior information to predict actions and others’ behaviors (Wolpert et al., 1998; Wolpert and Flanagan, 2001; Bastian, 2006; Roth et al., 2013). The model is updated in case of a mismatch between the prediction and sensorimotor feedback. Checking back could thus firstly be diagnostic of the violation of a social prediction, and secondly represent an attempt to update the model by retracking the gaze direction. These novel results indicate that the increased number of cerebellar neurons of birds likely allow for the formation of more stable internal forward models and the connected social predictions compared to other reptiles.

## The evolution of gaze following

The ability to visually co-orient with the gaze direction of others has been found in distantly related taxa, suggesting roots in deep evolutionary time. The origin of this skill, however, remains elusive. Perhaps, GFD evolved before vertebrates became land dwellers, or maybe shortly after. The lack of studies on non-amniotes, such as amphibians and fishes, makes it difficult to pinpoint the emergence of this skill. What speaks for a very old origin, is the conserved subcortical pathway in the vertebrate brain, involved in fast, reflexive co-orientation responses to the gaze of others. At least one fish species appears sensitive to body orientations of others, though this could be an adaptation to the species’ hunting style (see above). Based on evidence for GFD from all tested amniotes (mammals, reptiles, and birds), it is likely that this skill was present in the stem amniote, about 325 million years ago.

The ability for GGF, on the other hand, appears to have evolved in parallel, or convergently, in Synapsida (the lineage including the mammals) and Sauropsida (the lineage including the reptiles and birds), as this skill has to date only been found in mammals and birds. Decades of research in this area seem to have confirmed that GFD and GGF represent two distinct skills, as already suggested by Povinelli and Eddy (1996a). This implies that GGF relies on more complex, and hence later evolved, neurocognitive structures.

Two lines of evidence support this assumption. Firstly, in all species where the ontogeny of GGF has been studied, its onset clearly succeeds the development of GFD. Secondly, and perhaps more importantly, the two lineages exhibiting GGF skills, mammals and birds, have over time drastically increased their total and relative brain sizes, as well as their neuronal numbers. This disproportionally large increase left them with significantly more neurons relative to body size than reptiles. The heightened computational power connected to more neurons in the brain might equip mammals and birds with the capacity for visual perspective taking, while the lower neuronal numbers of reptiles do not allow for sophisticated visual socio-cognitive skills. However, more studies are needed to verify the absence of GGF in reptiles and to better understand the correlational relationship between neuronal numbers and GGF.

It is still unclear when GGF arose in the two different lineages. Mammals are the last extant representatives of the Synapsida. Thus, any comparisons with animal groups outside mammals, but within the synapsids, are not possible. However, within mammals, monotremes and marsupials have to our knowledge not been tested in gaze following. Monotremes are egg-laying mammals that diverged long before marsupial and placental mammals and are as close as we can get to the earliest mammals today. Marsupials are more derived, but their brains have retained more ancestral features compared to placental mammals (Ashwell, 2010; Álvarez-Carretero et al., 2022; Flannery et al., 2022; Kverková et al., 2022). Studies on these neurocognitively distinct groups would substantially support our understanding of the timing of the emergence of GGF in mammals, or to show that it might have even evolved before mammals.

In Sauropsida the picture is somewhat clearer. As GGF is not found in reptiles, it likely evolved in the dinosaur lineage. At least it seems to have existed in the first birds around 150 million years ago. But it is not unlikely that GGF existed in non-avian dinosaur taxa. The Maniraptora is the group of theropod dinosaurs from which the birds derived, and its members show overlapping traits with birds, in particular with palaeognaths. Their brains had morphologies comparable to modern palaeognaths (Balanoff et al., 2014). Moreover, they had comparable scaling relationships of body and brain size (Ksepka et al., 2020). Even some social behaviors connected to reproductive strategies were similar, such as the parental care system, where the male incubates the eggs from several females and provides care of the chicks (Varricchio et al., 2008; Varricchio and Jackson, 2016). That said, GGF might have been present even deeper into the non-avian dinosaurs. However, to better understand its origin, more studies on the neurocognition of GGF in birds are needed. Finally, more palaeontological neuroanatomy studies will help shed light on the evolution of GGF.

Gaze following—with its different levels—appears to be an important foundation for social cognition. This can, for example, be seen in the crucial role it plays for a developing human mind. Without gaze following a wealth of information is lost, and the opportunities to evolve essential skills, such as perspective taking and social predictions, are hampered. Considering the likely cardinal function this fundamental and underlying social behavior has, it is surprisingly understudied from an evolutionary perspective.

## Author contributions

CZ and TJ collected publications relevant for this review. CZ wrote the manuscript with extensive help of TJ and MO. All authors contributed to the article and approved the submitted version.

## Funding

This work was supported by Swedish Research Council grants 2021-02973 and 2019-03265, and the LMK Foundation. Open Access publishing fees have been covered by Lund University's APC fund.

## Conflict of interest

The authors declare that the research was conducted in the absence of any commercial or financial relationships that could be construed as a potential conflict of interest.

## Publisher’s note

All claims expressed in this article are solely those of the authors and do not necessarily represent those of their affiliated organizations, or those of the publisher, the editors and the reviewers. Any product that may be evaluated in this article, or claim that may be made by its manufacturer, is not guaranteed or endorsed by the publisher.
